# Historical development and current status of organ procurement from death-row prisoners in China

**DOI:** 10.1186/s12910-015-0074-0

**Published:** 2015-12-03

**Authors:** Kirk C. Allison, Arthur Caplan, Michael E. Shapiro, Charl Els, Norbert W. Paul, Huige Li

**Affiliations:** Program in Human Rights and Health/Division of Health Policy and Management, University of Minnesota School of Public Health of Minnesota, Minneapolis, USA; Division of Medical Ethics, Department of Population Health, New York University School of Medicine, New York, USA; Department of Surgery, New Jersey Medical School, Newark, USA; Department of Psychiatry, University of Alberta, Edmonton, Canada; Institute for History, Philosophy and Ethics of Medicine, Johannes Gutenberg University Medical Center, Mainz, Germany; Department of Pharmacology, Johannes Gutenberg University Medical Center, Obere Zahlbacher Strasse 67, 55131 Mainz, Germany

**Keywords:** Organ sourcing, Prisoners, Medical ethics, China

## Abstract

**Background:**

In December 2014, China announced that only voluntarily donated organs from citizens would be used for transplantation after January 1, 2015. Many medical professionals worldwide believe that China has stopped using organs from death-row prisoners.

**Discussion:**

In the present article, we briefly review the historical development of organ procurement from death-row prisoners in China and comprehensively analyze the social-political background and the legal basis of the announcement. The announcement was not accompanied by any change in organ sourcing legislations or regulations. As a fact, the use of prisoner organs remains legal in China. Even after January 2015, key Chinese transplant officials have repeatedly stated that death-row prisoners have the same right as regular citizens to “voluntarily donate” organs. This perpetuates an unethical organ procurement system in ongoing violation of international standards.

**Conclusions:**

Organ sourcing from death-row prisoners has not stopped in China. The 2014 announcement refers to the intention to stop the use of organs illegally harvested without the consent of the prisoners. Prisoner organs procured with “consent” are now simply labelled as “voluntarily donations from citizens”. The semantic switch may whitewash sourcing from both death-row prisoners and prisoners of conscience. China can gain credibility only by enacting new legislation prohibiting use of prisoner organs and by making its organ sourcing system open to international inspections. Until international ethical standards are transparently met, sanctions should remain.

**Electronic supplementary material:**

The online version of this article (doi:10.1186/s12910-015-0074-0) contains supplementary material, which is available to authorized users.

## Background

On December 3, 2014, Huang Jiefu, the director of the China Organ Donation Committee and former Vice-Minister of Health, announced that after January 1, 2015 only voluntarily donated organs would be used for transplantation in China. Media worldwide reacted optimistically, reporting that China would stop using executed prisoners as an organ source.

A few journalists, however, did question the credibility of the announcement. The Wall Street Journal, for example, noticed that “authorities haven’t publicly released details, so specific changes to China’s organ-donor laws aren’t clear” [1]. Christina Berndt, science editor of the Süddeutsche Zeitung [2], and Petra Kolonko, Beijing correspondent of the Frankfurter Allgemeine Zeitung [3], disclosed the “open back door” of the new rule: death-row prisoners are still allowed to “voluntarily donate” organs.

In short correspondence letters to The Lancet [4], we and others have pointed out that enthusiasm for an assumed shift under the announced policy does not reflect the reality in China [5–8]. History shows there is reason for concern, for remaining ethically vigilant, and for continued pressure for reform.

## Discussion

### Before 2005: decades of denying

China’s transplant medicine started with reliance on organ procurement from executed prisoners. The free donation rate in China was extremely low. The main reason was the lack of a cadaver organ donation system before 2010 and people had nowhere to donate, as recently admitted by Huang Jiefu [9]. From 1977 to 2009, a total of 130 deceased organ donations were reported [9, 10], which is in clear contrast to the approximately 120,000 total organs reported as transplanted in China during this same time period. Despite these facts, China denied the use of prisoner organs for decades until 2005.

#### “Profit-driven, unethical and violating human rights”

Organ harvesting from executed prisoners in China was based on informal arrangements between hospitals and the local courts that supervise the penal system. For decades, organs from executed prisoners were simply taken without consent. After the regulations of 1984 and 2007, written consent was officially required. However, this rule was largely ignored. In 2013, a Chinese medical official admitted that “previously, authorities used executed criminals’ organs without their consent, while permission has been required in recent years” [11].

Before 2007, hospitals in China did not need a transplantation license; any hospital could perform organ transplantation if it could obtain organs. With a burgeoning international transplant tourist market, the number of transplant hospitals increased from 150 before 1999 to over 600 in 2006 [12, 13]. Chinese media described the situation as “a chaos in which over 600 hospitals fought against each other for organ ‘donors’ and each hospital did things in its own way” [14] (for the Chinese original text see Additional file 1: Table S1).

There were well documented cases in which prisoners deliberately were not killed immediately in the execution (e.g. shooting not the head but the right chest instead) and organs were harvested from the still-living bodies in order to improve the quality of the extracted organs [15–17].

On several occasions, key officials admitted that the practice of organ harvesting from executed prisoners in China was “profit-driven, unethical and violating human rights” [18].

#### The 1984 regulation

In 1984, the first “Provisional Regulation on the Use of Dead Bodies or Organs from Condemned Criminals” was enacted directing the Supreme People’s Court, the Supreme People’s Procuratorate (office of prosecutor), Ministry of Public Security, Ministry of Justice, Ministry of Public Health and Ministry of Civil Affairs. This regulation officially permitted organ harvesting from prisoners executed expressly by shooting, if the body is not claimed; if the prisoner volunteers for organ donation; or if the family consents [19].

However, Huang Jiefu said in an interview with the China Youth Net in March 2015 that “the 1984 regulation was not a law; it was a secret regulation. I have never seen it; why are you able to see it? That is not a law. At the governmental level, our country has never officially recognized that the use of prisoner organs is legal. […] Why speaking again and again about abolishing it [the 1984 regulation]? Who has ever recognized that document?” [20] (for the Chinese original text see Additional file 1: Table S1).

The 1984 regulation was indeed somewhat unusual. It states that “the use of the dead bodies or organs from condemned criminals must be kept strictly confidential”. Nevertheless, the legal effect of this regulation is similar to that of departmental rules [21]. For the cessation of prisoner organ use, the 1984 Regulation must be explicitly abolished. The quasi-occult nature of the regulation has itself become an impediment to reversing course regarding prisoner organ sourcing.

### 2006-2014: stumbling forward

In December 2005, Huang admitted for the first time that organ sourcing from executed prisoners for transplantation was widespread with up to 95 % of the organ transplants in China deriving from executions.

After Huang’s acknowledgement, the Transplantation Society (TTS) expressed its objection to China’s practice of harvesting organs from executed prisoners through an academic embargo that prevents Chinese physicians who engage in this practice from presenting at international congresses, publishing articles in the medical literature, and achieving membership in TTS [22]. Huang Jiefu said in a recent interview that he is the only person from mainland China remaining a TTS member until today [20]. The reason why Huang is exempted from the TTS embargo is not clear.

In May 2006, the World Medical Association (WMA) demanded China to immediately cease the practice of using prisoners as organ sources [23]. However, China did not stop using prisoner organs. One rationale given was that a huge number of patients, circa 300,000, needed transplants each year. This number, however, proved to be unreliable.

#### Top secret: the number of patients waiting for organs in China

A Beijing Times reporter noticed in March 2014 that there were only 10,000 registered patients in China waiting for organ transplants [24]. The reporter asked Huang for the reason for the discrepancy between 10,000 and 300,000. Huang’s answer: “Organ transplantation in China requires a fee: a liver transplant or a kidney transplant costs hundreds of thousands of Yuan. Average folks cannot do it just because they want to. There is a large gap between those who really need and those who actually register. There are many reasons for their decision. Some seriously ill people need a transplant, but since the transplantation will exhaust all the money he has, he might give up. Such a situation is very common. Each year, one million people are on dialysis, about 1/3 of them need kidney or liver transplants. Therefore, by scientific estimation, about 300,000 will need transplants” [24]. Even after this interview, Huang continued to state that around 300,000 patients in China were in “urgent need” of organ transplants every year [25, 26].

In an interview with the Phoenix Satellite Television in March 2015, Huang indicated why he had operated with the wrong number, stating that currently 22,000 patients in mainland China were awaiting organ transplants adding “this is an accurate number”. The journalist was quite puzzled why before 2015 the real number was kept secret. Huang explained: “The death penalty is a state secret. Organs were sourced from executed prisoners. If you know the number of transplantations performed, then you would know the state secret”. The journalist reacted with “It can only be correct if the transplantation number is lower than the number of execution. There must be another reason”. Huang then said: “the issue you are talking about is too sensitive; that’s why I cannot tell you that clearly. If you think about it, you will understand. Because the country has no transparency, you don’t know how the organs were obtained; the number of performed transplantation was also a secret” [27, 28] (for the Chinese original text see Additional file 1: Table S1).

That organ transplantation and post-operational expense are not covered by medical insurance in China and thus are beyond the reach of a large number of medically indicated Chinese patients helped fuel international transplant tourism to China and the current domestic situation of “the poor donating organs while the rich getting transplanted”. This does not conform to World Health Organization (WHO) guiding principle 9 which demands that the allocation of organs “should be guided by clinical criteria and ethical norms, not financial or other considerations” [29].

#### Developments since 2006

In July 2006, a new rule was enacted, the Interim Provisions on the Administration of Clinical Application of Human Transplant Techniques. Ministry of Health spokesman Mao Qun’an characterized this as the “first time a Chinese health authority has set up a special committee and taken measures to help regulate organ transplants” [30].

In May 2007, a new law on organ transplantation (Regulation on Human Organ Transplantation) came into force, banning organ trading and the removal of a person’s organs without their prior written consent [31]. The 2007 regulation generically allows organ procurement, given that written consent is obtained. This applies equally to death-row prisoners, because there is no law in China that distinguishes prisoners from regular citizens for organ “donation”.

In the same year, the Ministry of Health drastically cut the number of transplant hospitals from over 600 by accrediting 164 to provide organ transplantation services. These hospitals were designated to function also as organ procurement organizations to administer organ donation and recovery [31].

In 2010, a pilot organ donation program was introduced in China [32] which evolved into a national program and distribution system - the China Organ Transplant Response System (COTRS) - in 2013 [31]. This is an important milestone, although the program still did not fully conform to international standards. WHO guiding principle 7 requires that donations must be “unpaid and truly voluntary” [29]. In China, however, significant financial compensation is provided making deceased organ donation attractive especially for economically vulnerable families [32].

#### Unfulfilled promises

In 2007, the year before the Olympic Summer Games in Beijing, the Chinese Medical Association (CMA) committed to end organ sourcing from prisoners in a letter to the WMA [33]. However, harvesting of prisoner organs did not stop.

On November 1, 2013, 38 hospitals signed an agreement (the so-called “Hangzhou Resolution”) to immediately stop using executed prisoners’ organs [34]. However, first-hand reports from China suggest that the practice of using prisoner organs continued also in a number of these hospitals [35]. In March 2014, Huang announced the plan to integrate prisoner organs into the COTRS voluntary organ donation system [24, 36]. This announcement indirectly confirmed that the Hangzhou Resolution had failed and the use of prisoner organs was continuing.

### 2015: a ticket to the future or the past?

As mentioned above, Huang announced in December 2014, that only voluntarily donated organs could be used for transplantation in China after January 1, 2015. What was most striking is that the announcement was not accompanied by any changes to China’s organ donation laws or governmental regulations. In fact, no plan of legal changes was announced, nor confirmatory governmental documents referenced, nor statements or comments from higher national health authorities cited. On the website of PRC National Health and Family Planning Commission no information could be found in English or Chinese related to the new announcement (http://en.nhfpc.gov.cn/regulations.html). The announcement of December 2014 itself is neither a law nor a governmental regulation. It is only at best a statement of good intentions but has no force of law.

Only recently in an interview with the China Youth Net, Huang did reveal the legal basis for the December 2014 announcement: “The first is the Regulation on Human Organ Transplantation issued in 2007, the second is the criminalization of organ trading in the Criminal Law Amendment issued in 2011, and the third important legislation is the Procurement and Allocation Regulations on Donated Human Organs issued by the National Health and Family Planning Commission in August 2013. These three documents prescribe that the source of the organ must be voluntary, unpaid, open, transparent and traceable. That is, knowing where the organ is from and where the organ is to go. According to this spirit, we announced the cessation of the use of organs from executed prisoners on January 1, 2015” [20] (for original Chinese text see Additional file 1: Table S1).

But how can the prior laws and regulations (2007–2013) be the basis for a new announcement in 2015? According to Huang, the problem with China’s organ transplantation system was that the laws were not obeyed: “Although the law provides that the use of prisoner organs must be from voluntary donation, but there are still loopholes in the law enforcement” [37]. This indicates that organs had also been procured without “consent” of prisoners, even by internationally noncompliant Chinese standards. “‘The rule of law’ was the 2014 iconic vocabulary in China” [37], and “2014 was the transitional test period for Chinese organ transplantation. […] The domestic and international environment, especially the climate of anti-corruption in China, has made it possible to announce it [the cessation of using organs from executed prisoners]” [27]. To translate Huang’s statements into simple words: The organ donation laws were largely ignored before 2014. The anti-corruption campaign and ‘the rule of law’ slogan in 2014 made Huang Jiefu believe that the laws and regulations would now be obeyed. Based on this assumption, Huang announced in December 2014 that only voluntarily donated organ would be used after 2015.

Huang’s statement makes clear that the new announcement is not based on new laws but on old existing ones. However, none of the three laws or regulations prohibits the use of prisoner organs; otherwise the practice of organ sourcing from prisoners between August 2013 and December 2014 (whether with or without “consent”) would have been illegal in toto. The three laws and regulations only prohibit forced organ harvesting from any person without consent. According to these, use of prisoner organs is still legal, as long as informed consent is provided. It should be noted that the international ethical standard, including that of the WMA and TTS, precludes prisoner organ sourcing in principle given the inherently coercive context of the prison environment [38, 39].

### Labelling prisoner organs as voluntary donations from citizens: the attempt to permanently legalize prisoner organs

The announcement by Huang on December 3, 2014, is unique. On the one hand, Huang stated that only voluntarily donated organs would be used for transplantation in China after January 1, 2015; yet on the other hand, he told reporters that “prisoners are still among the qualified candidates for donations” [36].

To understand these obviously contradictory statements, we need to look back 2 years. At a press conference on May 17, 2013, Huang criticized the practice of organ sourcing from executed prisoners [18]. But only 3 days later, he defended organ “donation” by death-row prisoners: “Before he [a death-row prisoner] died, he formed a conscience and found, they need to do something to repay the society [by donating organs]. So why you object?” (English original text) [16].

Huang seems to have re-invented the definition of prisoner organs. According to his definition, only organs sourced from prisoners in the “traditional way” (i.e. harvested without consent but through informal arrangement between hospital and local courts) are considered prisoner organs. If “consent” is provided, he classifies these organs as voluntary donations from citizens.

This became apparent in March 2014, when he announced a plan to integrate organs from executed prisoners into the existing voluntary organ donation and allocation system (COTRS) for “fair, transparent, and corruption-free” distribution [40]. The intention of this unprecedented step is clearly shown in Huang’s interview with the Beijing Times: “Death-row prisoners are also citizens and have the right to donate organs. The key is to have the regulations. From now on, death-row prisoner’s donation also requires the consent of the prisoner and the family members, which is the same as citizen donation. In the meantime, based on our country’s law, they [executed prisoners’ organs] will be enrolled into the computerized system for fair organ distribution. […] Once the organs from willing death-row prisoners are enrolled into our unified allocation system, they are then treated as voluntary donation from citizens; the so-called donation from death-row prisoners doesn’t exist any longer” [24] - a semantic trick [41].

In response to this unexpected development, TTS and the Declaration of Istanbul Custodian Group published an open letter to PRC president Xi Jinping calling on China to address its unethical practices in organ transplantation [22]. Meanwhile, international transplant leaders have called for a cessation of interaction with Chinese professionals until they stop using organs from executed prisoners [35]. Unfortunately, the overall international response has been insufficient. This may be the reason why Huang continued his course even after the announcement of December 3, 2014.

The China Daily quoted Huang on December 4, 2014: “Prisoners are still among the qualified candidates for donations, but their organs will be registered in the computerized system instead of being used for private trades, which will be the main difference in the future” [36]. A subsequent China Daily report on January 8, 2015 stated again that “prisoners will still qualify to donate, but their organs will be registered and put on the national database” [42].

In an interview with Phoenix Satellite Television on January 11, 2015, Huang added an astonishing claim: “we certainly do not want to use such words like ‘organ donations from death-row prisoners’. […] In their developmental process of organ transplantation, all countries in the world began with death-row prisoner donations. […] I’m not saying I oppose death-row prisoner donations. If the death-row prisoners are truly moved by their conscience, then it’s not impossible. But it must go through the citizen organ donation system, through the Red Cross, through the online computer system for a fair and equitable distribution. That’s transparent” [43].

Because critics have risen [35, 44], Huang spoke occasionally in a different tone for outside of China. In an interview published in English on January 18, 2015, Huang stated that “we do not support the proposal to include prisoners in the voluntary citizen-based allocation system, rather we support the complete cessation of utilizing organs from executed prisoners” [9] - as if the proposal were from someone else. The same sentence, however, can be found in a journal paper he submitted as early as on September 25, 2014 (accepted on December 14, 2014) [45]. Given his repeated statements in January 2015 promoting prisoner organ sourcing, it is less than clear whether Huang always means what he is saying or whether prisoner organs are used but not included in COTRS.

On January 28, 2015, Huang again told People’s Daily “death-row prisoners are also citizens. The law does not deprive them of the right to donate organs. If death-row prisoners are willing to atone for their crime by donating organs, they should be encouraged” [46].

With such repeated statements, Huang is sending a clear message to Chinese hospitals that organs from executed prisoners can still be used as long as written “consents” are provided, i.e. the laws are obeyed. Moreover, Huang’s invention for allowing prisoners to “donate” organs appears to be supported or accepted by other China’s transplant officials. In a Beijing Youth Daily report in March 2015, Zhuang Yiqiang, Deputy General Secretary of the Chinese Organ Transplantation Development Foundation, told journalists that “either death-row prisoners or ordinary people, all have the right to freely decide whether to donate organs or not. Death-row prisoners are also human beings. If he or she is willing to donate organs after death, of course, he or she should not be discriminated against by the society. But the prerequisite is, it must be out of one’s own will ’ [47] (for the Chinese original text see Additional file 1: Table S1). The statements of Huang Jiefu and Zhuang Yiqiang confirm again that the use of prisoner organs is still legal in China.

Thus, death-row prisoners are still allowed or even encouraged to “voluntarily” donate organs. This continues to violate international ethical standards. It is a fundamental principle in transplant medicine that organ donation must be made voluntarily which in turn requires autonomous, informed decision making. Even with informed “consent”, using organs from prisoners is not acceptable: prisoners are neither free from coercion nor always fully informed, nor able to freely consent, nor are their families [48]. The WMA states unmistakably in its policy that “in jurisdictions where the death penalty is practised, executed prisoners must not be considered as organ and/or tissue donors. While there may be individual cases where prisoners are acting voluntarily and free from pressure, it is impossible to put in place adequate safeguards to protect against coercion in all cases” [38]. Also TTS “is opposed to the recovery of organs and tissues from executed prisoners”, specifically “because of the restrictions in liberty in a prison environment, it is unlikely that prisoners are truly free to make independent decisions and thus an autonomous informed consent for donation cannot be obtained. Further, the financial incentive for recovering organs from executed prisoners may become an incentive to increase the number of such organs available for transplantation” - both of which apply in China [39].

If the new practice of labeling prisoner organs as voluntary donations were accepted by the international medical community, China would officially bypass, in deed alter, international ethics guidelines and the use of prisoner organs would become permanently legal. Moreover, the new practice may facilitate ongoing forced organ harvesting from prisoners of conscience as well. Since 2006, mounting evidence suggests that prisoners of conscience are killed for their organs in China with the brutally persecuted Buddhist practice, Falun Gong, among others, being the primary target (see European Parliament resolution of December 12, 2013 [49] and European Parliament workshop on “organ harvesting in China” of April 21, 2015 [50]). By re-defining prisoners as regular citizens for “voluntary” organ donation, China’s national organ donation system whitewashes the use of organs from both death-row prisoners and prisoners of conscience.

## Suggestions for a clear path forward

To enable and ensure the cessation of using prisoner organs for transplantation, the 1984 Regulation that permits the use of prisoner organs must be abolished. Meanwhile, a new law that explicitly prohibits the use of organs from any kind of prisoners must be issued.

To conform with WHO guiding principles, payment neither in direct nor in disguised forms (e.g. compensations) should be practiced. The costs for organ transplantation and the subsequent pharmacological treatment should be covered by health insurance for a fair organ allocation independent of the economic status of the patients.

In a survey performed in Guangzhou in 2005, 61.3 % of the university students were, on paper, willing to donate organs after death [51]. Among those who were opposed to cadaveric organ donation, 47.4 % and 35.9 % were concerned about (i) body integrity after death and (ii) inappropriate use of donated organs, respectively [51]. These results indicate that while elements of Confucian thought are culturally a significant barrier to organ donation, corruption in medical practices also represents a major factor. While more publicity and education programs are surely needed to promote voluntary organ donation in China, moreover, ending unethical organ harvesting practices will provide two-fold progress: meeting the criterion for the transplantation community to remove sanctions internationally and improving the image of transplant medicine in China domestically. This, in turn, may significantly increase the willingness within Chinese society for voluntary organ donation and free both from an addictive reliance on the organs of the executed thereby eliminating an implicit positive demand on execution. Finally, to demonstrate transparency, transplant registries should be made accessible for the public and the numbers of transplantations and executions, including past historical numbers, must be published. Decoupling transplantation from execution provides as well a double natural experiment concerning trends for both the former and the latter which, over time, have become a subject of concern not only beyond China.

## Conclusions

Organs from executed prisoners are still being used for transplantation in China. The likely differences after January 2015 are that written consents are obtained and these organs are now classified as voluntary donations from citizens, accepted notwithstanding the fundamentally coercive context. Whether these organs are included in COTRS is not clear.

Contrary to the sincere hope for ethical organ sourcing in China [52], the dream is yet a dream while prisoners remain at risk under the demand and ongoing medical exploitation of execution. China will gain credibility only by enacting legislation prohibiting the use of prisoner organs and by making its organ procurement system transparent to independent verifications and open to international inspections.

International sanctions (e.g. the TTS academic embargo) and dialogue with China have substantially contributed to the positive progress within China (e.g. development of the 2013 national donation program). Until a real cessation in prisoner organ use in China is achieved and verified, sanctions must remain both for the benefit of China and for the integrity of international ethical standards.

## Additional file

Additional file 1: Table S1. Quotes from Chinese sources used in the article.(PDF 391 kb)

## Abbreviations

WHO: World Health Organization
COTRS: China Organ Transplant Response System
TTS: the Transplantation Society
WMA: World Medical Association
