# Supplementary material for: Historical development and current status of organ procurement from death-row prisoners in China
Source: BMC Med Ethics. 2015 Dec 3;16:85. doi: 10.1186/s12910-015-0074-0 (PMC4668660; doi:10.1186/s12910-015-0074-0)
Supplement: Additional file 1: Table S1. — Quotes from Chinese sources used in the article.(PDF 391 kb) [file 12910_2015_74_MOESM1_ESM.pdf]

Supplement to

Historical development and current status of organ procurement from death-row prisoners in China

Kirk C Allison, Arthur Caplan, Michael E Shapiro, Charl Els, Norbert W Paul, Huige Li

Supplementary Table S1. Quotes from Chinese sources used in the article (according to appearance in the article)

Reference	Original Text in Chinese	English translation
Ref. 14<br>Xinhua-Net,<br>March 21, 2015	中国曾出现 **600 多家医院各自为政开展器官移植，争抢器官供体的混乱局面**。<br>http://www.qh.xinhuanet.com/2014-03/21/c_119877364.htm or<br>https://archive.is/kCrAF#selection-637.8-637.43	A chaos in which **over 600 hospitals fought against each other for organ 'donors'** and each hospital did things in his own way
Ref. 20<br><br>China Youth Net,<br>March 13, 2015	黄洁夫：看未来，**1984 年的文不是法，这个规定是秘密的，我也没有看到过，你们为什么能看到呢？那个不是法，国家在公开的层面上，在政府的层面上从来没有承认过利用死囚器官是合法的，那是难堪的一页，是饮鸩止渴，权宜之计。现在我们有阳光的，为什么老是要去废除什么呢？谁承认 1984 年的那个文件了**？<br>http://d.youth.cn/sk/201503/t20150313_6524457.htm (page 2) or<br>https://archive.is/wvPN3#selection-423.4-423.139	Huang Jiefu:<br>"The 1984 regulation was not a law; it was a secret regulation. I have never seen it; why are you able to see it? That is not a law. At the governmental level, our country has never officially recognized that the use of prisoner organs is legal. [...]<br>Why speaking again and again about abolishing it [the 1984 regulation]? Who has ever recognized that document"?
Ref. 24<br><br>Beijing Times,<br>March 4, 2014	京华时报：之前说每年有 30 万人需要器官移植，但实际登记等待移植的只有一万人，这之间的差距怎么理解？<br>黄洁夫：中间的差距涉及到供体来源、社会经济情况、取舍等多种情况，**现在我国器官移植是收费的，一个肝移植或者肾移植都需要花费几十万的费用，不是百姓想做就能做的，因此真正需要做的和实际登记做的有很大差距。人们的取舍有很多原因，有的人病重需要移植，但一旦移植会把家里的钱全部花光，他可能就会放弃，这种情况很常见。每年有 100 万人透析，约 1/3 需要肾移植和肝移植，科学估算有**	Huang Jiefu:<br>"Organ transplantation in China requires a fee: a liver transplant or a kidney transplant costs hundreds of thousands Yuan. **The average folks cannot do it just because they want to.** There is a big gap between those who really need and those who actually register. There are many reasons for their decision. Some seriously ill people need a transplant, but since the transplantation will exhaust all the money he has, he

	约 **30** 万人需要移植。<br>http://dailynews.sina.com/gb/chn/chnpolitics/phoenixtv/20140304/12205515629.html or<br>https://archive.is/BeVgr#selection-753.34-753.194	might give up. Such a situation is very common. Each year, one million people are on dialysis, about 1/3 of them need kidney or liver transplants. Therefore, by scientific estimation, about 300,000 will need transplants”.
Ref. 27<br>Phoenix Satellite Television,<br>March 16, 2015	黄洁夫：**死刑是国家的秘密**，是吗？<br>许戈辉：但是病患并不是秘密呀。这个对不起，我真的不懂。<br>黄洁夫：**你器官的来源是来自于死刑犯**。<br>许戈辉：是，就是器官的来源它可以是秘密，但是这个，就是等待这个也是秘密是吗？<br>黄洁夫：那你从死…，**不是那你能够做多少，那就不是都是知道你是国家的秘密了吗**？<br>许戈辉：那它应该小于这个数字才对呀，还有一个原因就是。<br>黄洁夫：**你说这个太敏感，所以我不能跟你讲得太清楚，你一想就想清楚了，一定这个事情，因为你国家没有个透明的体现，这怎么来的你也不知道，做多少也是秘密**<br>http://news.ifeng.com/a/20150316/43349299_0.shtml or<br>https://archive.is/YRmiK#selection-793.0-817.103	Huang Jiefu.<br>“**The death penalty is a state secret**”.<br>“Organs were sourced from executed prisoners”.<br>“**If you know the number of transplantation performed, then you would know the state secret**”.<br>The journalist reacted “It can only be correct if the transplantation number is lower than the number of execution. There must be another reason”.<br>Huang then said: “**the issue you are talking about is too sensitive, that’s why I cannot tell you that clear**. If you think about it, you will understand. **Because the country has no transparency, you don’t know how the organs were obtained; the number of performed transplantation was also a secret**”.
Ref. 20<br>China Youth Net,<br>March 13, 2015	黄洁夫：<br>在这样的情况下，根据依法治国，根据什么法？**一个是根据 2007 年的《人体器官移植条例》，第二是根据 2011 年刑法修正案中的器官买卖罪，第三个很重要的法规是 2013 年 8 月份，卫计委下发的《人体捐献器官获取和分配管理规定》。这三个文件就规定了器官的来源必须是自愿、无偿，同时必须是公开、透明、可溯源的，就是知道从哪儿来，到哪儿去。根据这样的精神，今年我们就宣布了 2015 年 1 月 1 号取消死囚器官的使用。**<br>http://d.youth.cn/sk/201503/t20150313_6524457.htm or<br>https://archive.is/jfCbx#selection-379.2-379.199	Huang Jiefu:<br>“The first is the **Regulation on Human Organ Transplantation issued in 2007**, the second is the crimination of organ trading in the **Criminal Law Amendment issued in 2011**, and the third important legislation is the **Procurement and Allocation Regulations on Donated Human Organs** issued by the National Health and Family Planning Commission in **August 2013**. These three documents prescribe that the source of the organ must be voluntary, unpaid, open, transparent and traceable. That is, knowing where the organ is from and where the organ is to go. **According to this spirit, we announced the cessation of the use of organs from executed prisoners on January 1, 2015**”.

Ref. 24<br>Beijing Times,<br>March 4, 2014	黄洁夫：<br>**死囚也是公民，也有捐献器官的权利**，关键是有法治的建设，**今后，死囚器官捐献也将需要本人和家人同意，就和公民捐献一样，同时要按照国家法律，进入全国统一的计算机系统自动公平分配**。…<br>**有捐献意愿的死囚的器官一旦纳入我国统一的分配系统，就属于公民自愿捐献，不再存在死囚捐献的说法**。<br>http://dailynews.sina.com/gb/chn/chnpolitics/phoenixtv/20140304/12205515629.html or https://archive.is/BeVgr#selection-705.64-713.86	Huang Jiefu:<br>**"Death-row prisoners are also citizens and have the right to donate organs.** The key is to have the regulations. **From now on, death-row prisoner's donation also requires the consent of the prisoner and the family members**, which is the same as citizen donation. In the meantime, based on our country's law, **they [executed prisoners' organs] will be enrolled into the computerized system for fair organ distribution.** [...]<br>**Once the organs from willing death-row prisoners are enrolled into our unified allocation system, they are then treated as voluntary donation from citizens; the so-called donation from death-row prisoners doesn't exist any longer"**.
Ref. 37<br>CCTV News,<br>Jan 12, 2015	依法治国，是 2014 年的标志性词汇。…<br>在黄洁夫看来，器官捐献领域最大的有法不依就是针对死囚的器官使用问题。长期以来，中国器官移植来源主要依靠死囚，**尽管法律规定死囚应"自愿捐献器官"，但是法律执行上仍存在漏洞**。<br>http://m.thepaper.cn/newsDetail_forward_1293555 or<br>https://archive.is/s9I1c#selection-101.2-121.87	Huang Jiefu:<br>"**Although the law provides that the use of prisoner organs must from voluntary donation, but there are still loopholes in the law enforcement**".
Ref.27<br>Phoenix Satellite Television,<br>March 16, 2015	黄洁夫：2014 年，是中国器官移植中间接受考验的一年… 所以这个是国际国内的环境，特别是我们国内反腐倡廉的大的气候，才能够使我们能够宣布这一点。<br>http://news.ifeng.com/a/20150316/43349299_0.shtml or<br>https://archive.is/YRmiK#selection-879.0-879.146	Huang Jiefu:<br>"**2014 was the transitional test period for Chinese organ transplantation**. [...] The domestic and international environment, especially the climate of anti-corruption in China, has made it possible to announce it [the cessation of using organs from executed prisoners]".

Ref. 43<br>Phoenix Satellite Television,<br>Jan 11, 2015	黄洁夫：我们一定是不要用死囚捐献这几个字，因为在世界各个国家器官移植发展过程之中，都曾经有过死囚捐献的开始，但这些国家和地区一旦建立了公民捐献体制，就取消了死囚。我们必须得依靠公民自愿捐献为我们器官的来源，这样才能去谈你是一个阳光的伦理学的器官移植的世界，我不是说反对死囚捐献，死囚他真的良心发现了那未尝不可，但是必须是公民捐献的体系，通过红十字会，通过网上电脑上公平公正的分配，那是很阳光的<br>http://news.ifeng.com/a/20150111/42906812_0.shtml or<br>https://archive.is/YxJwf#selection-919.0-919.211	Huang Jiefu:<br>"**We certainly do not want to use such words like 'organ donations from death-row prisoners'**. [...] In their developmental process of organ transplantation, all countries in the world began with death-row prisoner donations. [...] **I'm not saying I oppose death-row prisoner donations. If the death-row prisoners are truly moved by their conscience, then it's not impossible. But it must go through the citizen organ donation system, through the Red Cross, through the online computer system for a fair and equitable distribution.** That's transparent".
Ref. 46<br>People's Daily,<br>Jan 28, 2015	中国器官捐献与移植委员会主任委员黄洁夫认为，**死囚也是公民，法律并未剥夺其捐献器官的权利。如果死囚愿意捐献器官赎罪，应该鼓励**。<br>http://society.people.com.cn/n/2015/0128/c1008-26461244.html or<br>https://archive.is/zc8bf#selection-627.3-627.65	Huang Jiefu:<br>"**Death-row prisoners are also citizens. The law does not deprive them of the right to donate organs. If death-row prisoners are willing to atone for their crime by donating organs, they should be encouraged**".
Ref. 46<br>Beijing Youth Daily,<br>March 4, 2015	中國器官移植發展基金會副秘書長莊一強：無論是死囚，還是普通人，都擁有自行決定是否捐獻器官的權利。死囚也是人，如果他（她）願意死後捐出器官，社會當然不該有所歧視。但是其前提，一定是"出於自願"。<br>http://big5.ce.cn/gate/big5/wap.ce.cn/szsh/201503/04/t20150304_4719641.html or<br>https://archive.is/Jvguy#selection-291.2-291.83	Zhuang Yiqiang<br>(Deputy General Secretary of the Chinese Organ Transplantation Development Foundation):<br>"**Either death-row prisoners or ordinary people, all have the right to freely decide whether to donate organs or not. Death-row prisoners are also human beings. If he or she is willing to donate organs after death, of course, he or she should not be discriminated by the society.** But the prerequisite is, it must be out of one's own will".
